# Analysis of 4 imaging features in patients with COVID-19

**DOI:** 10.1186/s12880-020-00484-1

**Published:** 2020-07-23

**Authors:** Jun Jin, De-hong Gao, Xin Mo, Si-ping Tan, Zhen-xia Kou, Yi-bo Chen, Jin-bo Cao, Wen-jing Chen, Ya-ming Zhang, Bing-qing Li, Kuan-long Huang, Bing-ren Xu, Xiao-li Tang, Yu-li Wang

**Affiliations:** 1 Department of Radiology, Shenzhen Nanshan District Shekou People’s Hospital, Shenzhen, 518067 Guangdong Province China; 2 Concorde Shenzhen Hospital, Huazhong University of Science and Technology, Shenzhen, 518052 Guangdong Province China; 3 Gansu Province Center for Disease Control and Prevention, LanZhou, 73000 Gansu Province China; 4 Department of Imaging, Shenzhen Second People’s Hospital / the First Affiliated Hospital of Shenzhen University, Shenzhen, 518035 Guangdong Province China

**Keywords:** Coronavirus disease 2019, imaging features, “feather sign”, “dandelion sign”, “pomegranate sign”, “rime sign”

## Abstract

**Background:**

The aim of this was to analyze 4 chest CT imaging features of patients with coronavirus disease 2019 (COVID-19) in Shenzhen, China so as to improve the diagnosis of COVID-19.

**Methods:**

Chest CT of 34 patients with COVID-19 confirmed by the nucleic acid test (NAT) were retrospectively analyzed. Analyses were performed to investigate the pathological basis of four imaging features(“feather sign”,“dandelion sign”,“pomegranate sign”, and “rime sign”) and to summarize the follow-up results.

**Results:**

There were 22 patients (65.2%) with typical “feather sign”and 18 (52.9%) with “dandelion sign”, while few patients had “pomegranate sign” and “rime sign”. The “feather sign” and “dandelion sign” were composed of stripe or round ground-glass opacity (GGO), thickened blood vessels, and small-thickened interlobular septa. The “pomegranate sign” was characterized as follows: the increased range of GGO, the significant thickening of the interlobular septum, complicated with a small amount of punctate alveolar hemorrhage. The “rime sign” was characterized by numerous alveolar edemas. Microscopically, the wall thickening, small vascular proliferation, luminal stenosis, and occlusion, accompanied by interstitial infiltration of inflammatory cells, as well as numerous pulmonary interstitial fibrosis and partial hyaline degeneration were observed. Repeated chest CT revealed the mediastinal lymphadenectasis in one patient. Re-examination of the NAT showed another positive anal swab in two patients.

**Conclusion:**

“Feather sign” and “dandelion sign” were typical chest CT features in patients withCOVID-19; “pomegranate sign” was an atypical feature, and “rime sign” was a severe feature. In clinical work, accurate identification of various chest CT signs can help to improve the diagnostic accuracy of COVID-19 and reduce the misdiagnosis or missed diagnosis rate.

## Background

Since the COVID-19 outbreak in December 2019, the number of confirmed cases has been rapidly increasing. So far (March 15, 2020), COVID-19, has spread to 146 countries and territories across six continents, infecting more than 164,000 and killing more than 6,400 people. Currently, COVID-19 is considered one of the worst epidemics in human history.

The COVID-19 is a lineage B betacoronavirus. According to the internationally published virus classification, there are nine types of this betacoronavirus [1], among which mouse hepatitis virus, rousettus bat coronavirus HKU9, and severe acute respiratory syndrome (SARS) are the most widely known. On February 11, 2020, the International Committee on Taxonomy of Viruses named COVID-19 as severe acute respiratory syndrome coronavirus 2 (SARS-CoV-2) [2]. Although treatment for.

SARS-CoV-2 is still not available, researchers are currently working on creating vaccines and investigating clinical features of the infected population.

Recently, Wang et al examined the CT images of asymptomatic infected patients with COVID-19 and found that chest CT scans have an essential role in the screening of the population suspected of having infection [3]. Besides, a previous report suggested that CT imaging may be very useful in the diagnosis of COVID-19 in patients with negative NAT [4]. In the present study, we analyzed clinical data and CT images of 43 COVID-19. These data could contribute to timely and accurate identification of the clinical features, laboratory test results , and CT imaging findings of COVID-19, thereby resulting in early diagnosis, quarantine, and treatment.

## Methods

### Patients

This study had no potential risks for patients, and there was no direct relationship between researchers and patients. The study was conducted according to the principles of the Helsinki Declaration. The ethics committee of Shekou people's Hospital waived the signing of informed consent for this retrospective study. Patients with laboratory-confirmed COVID-19 (confirmed by a reverse transcription polymerase chain reaction, RT-PCR) were recruited from three hospitals between January 22, 2020 and February 26, 2020. The number of cases included from each hospital is shown in Supplementary Table 1. Age, gender, epidemiological features, and clinical symptoms were collected from all patients.

### Chest CT and image analysis

All patients underwent a chest CT scan, which was performed using GE 256-row Revolution CT, GE Light Speed 16-slice spiral CT, Siemens SOMATOM Emotion 16-row spiral CT, and GE 64-row VCT, all of which were end-inspiratory scans. For the axial-section, the slice thickness was 5mm, and the reconstruction slice thickness was 1.25 or 0.625 mm. Two radiologists (with more than ten years of work experience) analyzed all images. Five patients who received chest radiography and four with normal chest CT images were excluded. Finally, 34 patients were included in the study.

**Fig. 1:**
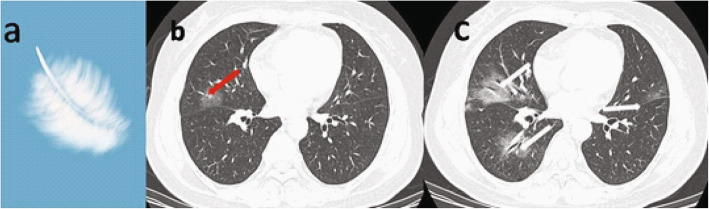
A older female patient with a long residence history in an epidemic area experienced fever(38.5 °C) and sore muscle for 1 day. **a** Feather hand painting. **b** Baseline chest CT image demonstrated GGO in the right middle lobe, showing a “feathery sign” (red arrow). The nucleic acid test was negative for the first time. **c** Follow-up CT scan after 6 days showed that the lesions were significantly enlarged. GGO in the lungs was multiple (white arrow). The second test was positive for nucleic acid

**Fig. 2:**
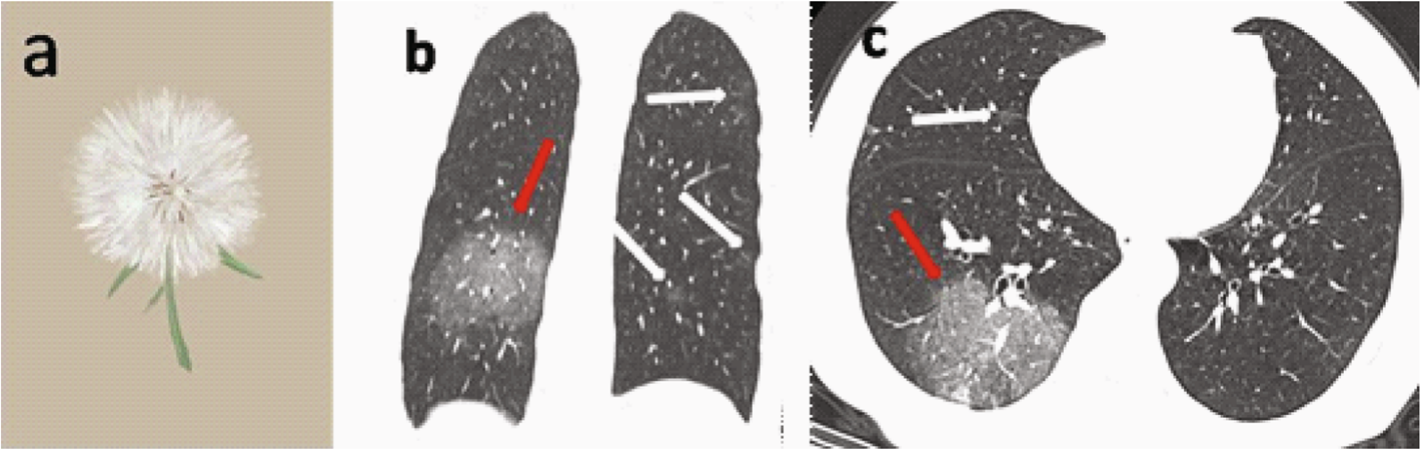
A older female patient with a long history of living in an epidemic area experiencing fever and sore throat for 4 days. **a** Dandelion hand painting. **b** Non-contrast enhanced coronal CT image shows that the right lower lobe with a circular GGO,vascular thickening, and bronchiectasis, showing a “dandelion sign” (red arrow), and multiple small patchy GGO in the left lung (White arrow). **c** Axial thin-section un-enhanced CT image shows a round-like GGO (red arrow) in the right lower lobe, a small piece of GGO in the right middle lobe, and the unclear border (white arrow)

**Fig. 3:**
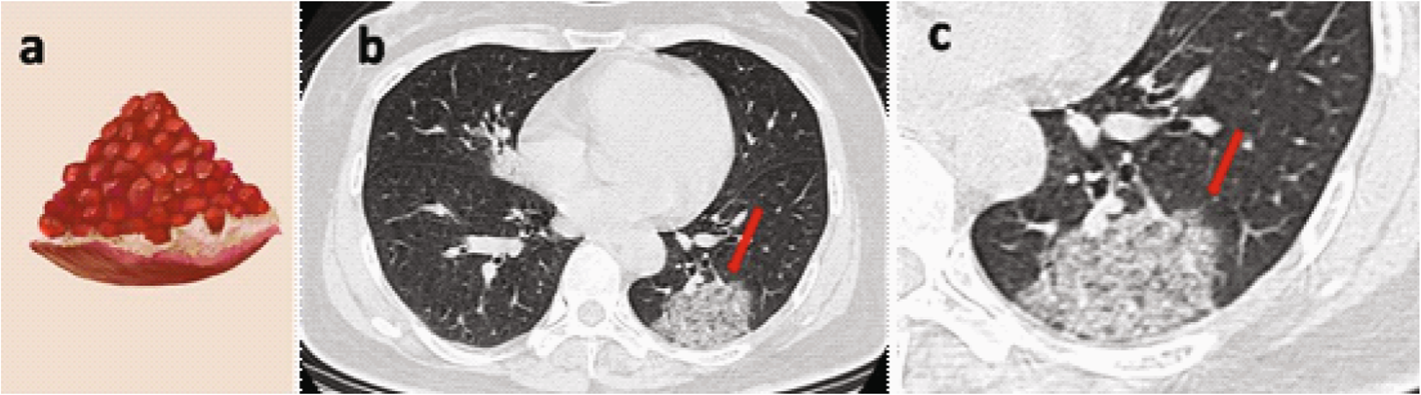
A older female patient experiencing fever and cough for 1 day,,and who had 3 days of travel history in the epidemic area before onset. **a** Pomegranate hand painting. **b** Non-contrast enhanced chest CT scan shows that the GGO in the posterior basal segment of the left lower lobe and vascular thickening, bronchiectasis, and interlobular septal thickening, showing a “pomegranate sign” (red arrow). **c** A partially enlarged image at the same level as in figure b, suggesting that the GGO in the posterior basal segment of the left lower lobe showed a “pomegranate sign” (red arrow)

**Fig. 4:**
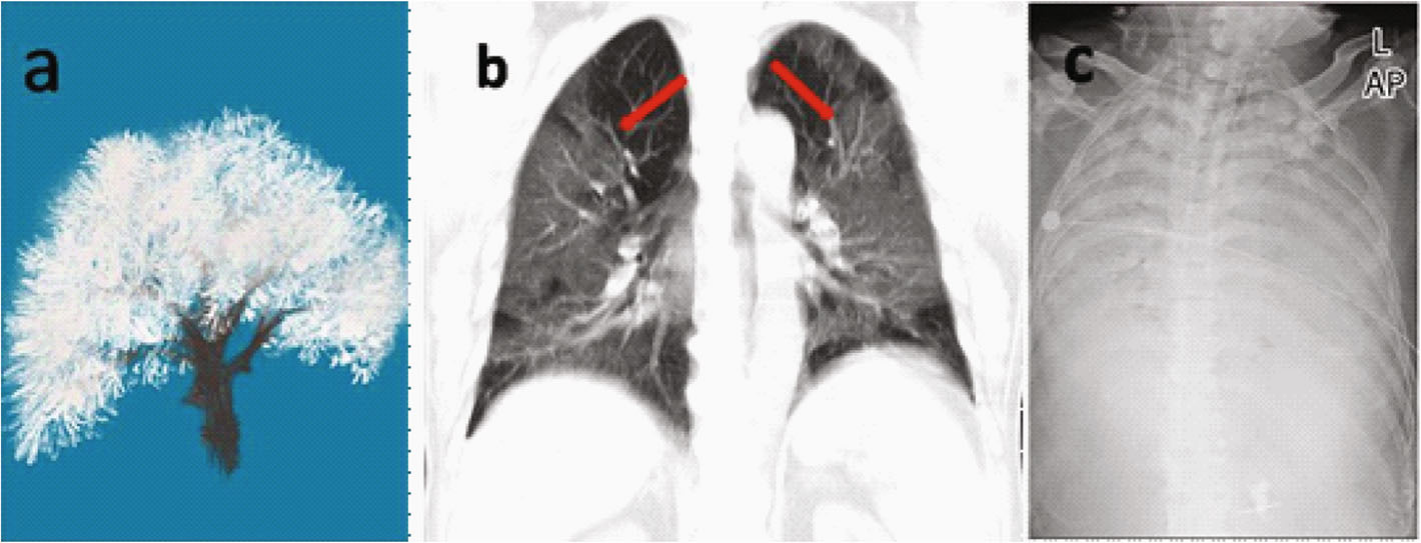
A older male patient who experienced constipation, and anorexia lasting for 1 week, and who had no epidemiological history. **a** Rime hand painting. **b** Coronal CT image of the chest, showing diffuse GGO in the lungs, vascular thickening, interlobular septal thickening, showing “rime sign” (red arrow). **c** On the 20th day after admission, bedside portable chest radiograph showed diffuse high-density shadows in both lung fields, and the lesions significantly progressed compared with the previous ones

The following CT image features were observed for each patient: (a) the location, extent, and a number of lesions; (b) type of lesions (GGO, vascular thickening, pulmonary consolidation, pulmonary fibrous, interlobular septum, and solid nodules); (c) specific signs (“air bronchogram sign”, “feather sign”, “dandelion sign”, “pomegranate sign”, “rime sign”); (d) other signs (pleural effusion, mediastinal lymphadenectasis,etc). The CT images of solid nodule and pulmonary consolidation showed the density of lesions covering the vascular and bronchial shadows resembling [5]; “feather sign” or “dandelion sign” ,which was defined as the exudative lesion and thickened blood vessels forming a strip or round high-density shadow, which was very similar to the shape of feathers or dandelions (Figs. 1 and 2); “pomegranate sign”, which was defined as an exudative lesion accompanied by a small amount of bleeding, showing round and imbricate arrangement that was similar to a pomegranate (Fig. 3); “Rime sign”, which was defined as multiple exudative and punctate hemorrhage in the lesion accompanied with extensive interstitial fibrosis forming large white lung, that was similar to white rime attached to the branches (Fig. 4).

Fourteen out of 30 patients underwent chest CT re-examination 5 to 14 days after being cured. Two senior radiologists compared CT images for two or more times. The remaining 16patients did not undergo CT scanning due to the quarantine period.

## Results

There were 43 patients in this group, including 20 males (46.5%) and 23 females (53.5%). Predominantly, these were middle-aged and elderly patients, with a mean age of 52.4±14.8 years (range, 19-78 years). Patient characteristics are showed in Table 1.

**Table 1:** Patient characteristics

	Patients (***n*** = 43)
**Patient demographics**	
Median age, years (range)	52.4 ± 14.8 (19–78)
Men	20(46.5%)
Women	23(53.5%)
**Exposure history**	
Exposure to epidemic area	36(83.7%)
Unknown exposure	7(16.3%)
**Signs and symptoms**	
Fever	41(95.3%)
Chest distress	2(4.7%)
Cough	23(53.5%)
Weak	10(23.3%)
Headache	2(4.7%)
Sore muscle	8(18.6%)
Gastrointestinal discomfort	8(18.6%)
Expectoration	6(14.0%)
Sore throat	6(14.0%)
Pharyngeal congestion	4(9.3%)
Dizziness	3(7.0%)
Chills	8(18.6%)

A baseline chest CT scan was abnormal in 34 patients (Table 2); 23 (67.6%) showed lesions involving both lungs, and 24 (70.6%) reported bilateral multifocal lesions, with the predominant lower lobe. In addition, there were 29 patients (85.3%) with GGO and vascular thickening in the lesion, 16 (47.1%) with air bronchogram sign, 29 (85.3%) with interlobular septal thickening, 22 (65.2%) with “feather signs”, 18 (52.9%) with “dandelion sign” and 21 (61.8%) with pulmonary fibrous tissue proliferation. The partial consolidation of the lesion, solid nodules, “pomegranate sign”, and “rime sign” were rare. One (2.9%) patient had pleural effusion and mediastinal lymphadenectasis. Also, only five patients underwent chest X-ray; two showed a positive result for X-ray; four patients reported negative results for chest CT scan, and two of them showed multiple GGO during re-examination after three days.

**Table 2:** Imaging findings of patients with COVID-19

	Patients (n=34)
**Area of lession**	
Unilateral lung	11(32.4%)
Bilateral lung	23(67.6%)
**Number of lession**	
single lession	10(29.4%)
Multiple lessions	24(70.6%)
**Lobe of lesion distribution**	
Multiple lobes	19(55.9%)
Two lobes	4(11.8%)
One lobe	11(32.3%)
**Vascular thickening**	
Yes	29(85.3%)
None	5(14.7%)
**Air bronchogram sign**	
Yes	16(47.1%)
None	18(52.9%)
**GGO**	
Yes	29(85.3%)
None	5 (14.7%)
**Pulmonary consolidation**	
Yes	8(23.5%)
None	26(76.5%)
**Pulmonary fibrosis**	
Yes	21(61.8%)
None	13(38.2%)
**Interlobular septal thickening**	
Yes	29(85.3%)
None	5(14.7%)
**“feathery sign”**	
Yes	22(65.2%)
None	12 (34.8%)
**“Dandelion sign”**	
Yes	18(52.9%)
None	16(47.1%)
**“Pomegranate sign”**	
Yes	9(26.1%)
None	25(73.9.%)
**“rime sign”**	
Yes	7(20.6%)
None	27(79.4%)
**Solid nodule**	
Yes	3(8.8%)
None	31(91.2%)
**Pleural effusion**	
Yes	1(2.9%)
None	33(97.1%)
**Mediastinal Lymphadenopathy**	
Yes	1(2.9%)
None	33(97.1%)

All patients were followed up. Among the 13 hospitalized patients, two showed rapid progression during the hospital stay and were in critical condition. Thirty 30patients were cured and discharged. Four patients had cough and chest distress on re-examination after 5-14 days. Laboratory re-examination revealed that four patients had elevated T-lymphocyte counts, accompanied by elevated alanine aminotransferase and creatinine levels. CT re-examination indicated the following: two patients had no obvious changes; eight reported improved absorption; four reported that lesions were completely absorbed, and one had mediastinal lymphadenectasis. NAT was performed in 14patients. Two patients were positive for anal swabs, while they were negative for nasal and throat swabs; the remaining 12 patients were normal, as shown in Table 3.

**Table 3:** Follow up 5–14 days after discharge

	Patients (***n*** = 14)
**The mean hospitalization days**	21
**Signs and symptoms**	
Cough	4
Chest distress	4
Weak	2
Shortness of breath	2
Stomachache	4
**Laboratory test**	
T lymphocyte count increased	4
T helper cell count increased	4
Cytotoxic T lymphocyte count increased	4
Total bilirubin increased	4
Indirect bilirubin increased	2
Alanine aminotransferase increased	4
Glutamic oxaloacetylase increased	4
Greatinine increased	4
**Chest CT**	
No change	2
Improved absorption	8
Resolution	4
**Mediastinal Lymphadenopathy**	
None	13
Yes	1
**Nucleic acid test by Real-time PCR**	
Negative	12
Positive	2

## Discussion

In the present study, we found that the most common CT imaging features in patients with COVID-19 were: bilateral, multifocal GGO, peripheral distribution; the predominant lower lobe; pleural effusion and lymphadenectasis were rare, which is consistent with previous reports [6–8]. In addition, “feather sign”was found in 22 patients (65.2%), “dandelion sign” in 18 (52.9%), “pomegranate sign” in nine (26.1%), and “rime sign” in seven (20.6%) patients, which could be considered as new features in patients with COVID-19. The “feather sign” and “dandelion sign” on the CT image included stripe or round GGO, thickened blood vessels, and small-thickened interlobular septa. GGO shows diffuse alveolar damage under the microscope, which is histologically caused by alveoli filled with blood, pus, water, or cells [9, 10]. The reason for the thickening of blood vessels in the lesion may be the following:under the effect of inflammatory factors, the increased permeability of the vascular wall may lead to the dilation of capillaries and the corresponding thickening of the pulmonary artery [11]. The incidence of “feather sign” and “dandelion sign” in this study was 65.2% and 52.9%, respectively.

In this study, nine patients (26.1%) presented with “pomegranate sign”, which is an atypical chest CT feature of COVID-19. A “pomegranate sign” can be characterized as a further increase of the range of ground-glass opacity that occupies part of the lung sub-segment, the more significant thickening of the interlobular septum, complicated with a small amount of punctate alveolar hemorrhage [9, 12], and lesions that are in imbricate arrangement, and are similar to a pomegranate. Moreover, among seven patients (20.6%) who developed a “rime sign”, two were critically ill. A “rime sign” is characterized by numerous alveolar edemas. Hemorrhagic necrosis can be observed in some alveoli. Moreover, mucus and hemorrhagic exudate diffusely cover the bronchiole wall. Microscopically, the wall thickening, small vascular proliferation, luminal stenosis, and occlusion, accompanied by interstitial infiltration of inflammatory cells, such as lymphocytes, plasma cells, and monocytes [12], as well as numerous pulmonary interstitial fibrosis and partial hyaline degeneration are observed. This type of lesion has a wide range and looks like a white rime attached to abranch.

All patients were followed up for two weeks. Among 13 hospitalized patients, 11 had stable conditions and gradually recovered, two reported rapid progression during the hospital stay and were in critical condition, and underwent extracorporeal membrane oxygenation (ECMO). Thirty patients were cured and discharged. Fourteen underwent re-examination after 5-14 days;four had a cough, stomachache and chest distress. Very few patients developed weakness and shortness of breath. Laboratory tests revealed a significant increase in T-lymphocyte counts in four patients (flow cytometry: 762/ul, 899 /ul, respectively; the normal value was 0), accompanied by a significant increase in absolute counts of helper and cytotoxic T-lymphocytes, while alanine aminotransferase, creatinine and total bilirubin levels were increased to varying degrees, which suggested that the patient's immune function was deteriorated, and liver and renal functions were impaired; these data were consistent with previous reports [13]. These findings suggest that liver injury may be caused by SARS-CoV-2 infection or induced by drug treatment during hospitalization.

Among patients who underwent CT re-examination, two patients showed no obvious changes; in four patients, lesions were completely absorbed; in eight cases, lesions were partially absorbed; and one had mediastinal lymphadenectasis. These data suggest that CT can be used to monitor changes during disease progression, which is consistent with the findings of Hosseiny et al [14].

Multiple NAT (including nasal, anal, and throat swabs) were performed after 5-14 days. In two patients, the results of NAT of nasal and throat swabs were negative, while the result of NAT of the anal swab was positive, which is why the patients were immediately readmitted to the hospital for treatment. The remaining 12patients reported negative results for multiple NAT. A recent study [15] revealed that four patients with COVID-19 showed “positive results” for nucleic acid test 5-13 days after discharge. This suggested that current discharge standards should be revised; nasal, anal, and throat swabs should be combined, as well as supplemented by a variety of other testing methods. COVID-19 patients should be monitored during treatment, rehabilitation, and quarantine, so as to fundamentally control the occurrence of the “positive results” after discharge [16].

This study had some limitations. Firstly, no children are enrolled in this study, and the clinical, epidemiological, and imaging features of children with COVID-19 are lacking. Secondly,The number of patients collected in this study is so small that study results have certain limitations. The reliability of the conclusion needs to be further expanded to verify the sample size. Thirdly, sufficient pathological specimens are currently unavailable for comparison with imaging features. We will collect more patients data and pathological specimens to observe the evolution and outcome of the disease and determine the correlation between the imaging and pathology.

## Conclusion

Our data suggested that “feather sign” and “dandelion sign” were typical chest CT features of COVID-19. “Pomegranate sign” was an atypical feature, and “rime sign” was a severe feature, which suggested poor prognosis. In clinical work, accurate identification of various chest CT signs in combination with epidemiological history, clinical features, multiple nucleic acid tests, and other testing methods can help improve the diagnostic accuracy of COVID-19 and reduce the misdiagnosis or missed diagnosis rate.

## Supplementary information

**Additional file 1.**

## Data Availability

The datasets used and analyzed during the current study are available from the corresponding author on reasonable request.

## Abbreviations

COVID-19: Coronavirus Disease 2019
RT-PCR: Reverse Transcription Polymerase Chain Reaction
NAT: Nucleic acid test
GGO: Ground-glass opacity
