# Supplementary material for: Analysis of 4 imaging features in patients with COVID-19
Source: BMC Med Imaging. 2020 Jul 23;20:84. doi: 10.1186/s12880-020-00484-1 (PMC7376520; doi:10.1186/s12880-020-00484-1)
Supplement: Supplementary file 1 — Additional file 1. [file 12880_2020_484_MOESM1_ESM.docx]

Supplementary Table 1. A list of participating hospitals with contributed number of patinets

Hospital Location COVID-19 Cases

Shekou People’s Hospital of Shenzhen Guangdong Province, China 9

the Second People's Hospital of Shenzhen Guangdong Province, China 16

Concorde Shenzhen Hospital, Huazhong University of Science and Technology

Guangdong Province, China 18
